# Regional differences in the predictors of acute electrical reconnection following high‐power pulmonary vein isolation for paroxysmal atrial fibrillation

**DOI:** 10.1002/joa3.12597

**Published:** 2021-07-23

**Authors:** Kyoichiro Yazaki, Koichiro Ejima, Shohei Kataoka, Miwa Kanai, Satoshi Higuchi, Daigo Yagishita, Morio Shoda, Nobuhisa Hagiwara

**Affiliations:** ^1^ Department of Cardiology Tokyo Women's Medical University Tokyo Japan; ^2^ Clinical Research Division for Heart Rhythm Management Department of Cardiology Tokyo Women's Medical University Tokyo Japan

**Keywords:** acute pulmonary vein reconnection, atrial fibrillation, high‐power pulmonary vein isolation, impedance drop, unipolar signal modification

## Abstract

**Background:**

Acute pulmonary vein reconnection (PVR) is associated with long procedure times and large radiofrequency (RF) energy delivery during pulmonary vein isolation (PVI). Although the efficacy of high‐power PVI (HP‐PVI) has been recently established, the determinants of acute PVR following HP‐PVI remain unclear.

**Methods:**

We evaluated data on 62 patients with paroxysmal atrial fibrillation undergoing unipolar signal modification (USM)‐guided HP‐PVI. A 50‐W RF wave was applied for 3‐5 seconds after USM. In the segments adjacent to the esophagus (SAEs), the RF time was limited to 5 seconds. Each circle was subdivided into six regions (segments), and the possible predictors of acute PVR, including minimum contact force (CF_min_), minimum force‐time integral (FTI_min_), minimum ablation index (AI_min_), minimum impedance drop (Imp‐min), and maximum inter‐lesion distance (ILD_max_), were assessed in each segment.

**Results:**

We investigated 1162 ablations in 744 segments (including 124 SAEs). Acute PVR was observed in 21 (17%) SAEs and 43 (7%) other segments (*P* = .001). The acute PVR segments were characterized by significantly lower CF_min_, FTI_min_, AI_min_, and Imp‐min values in the segments other than the SAEs and larger ILD_max_ values in the SAEs. Furthermore, lower Imp‐min and larger ILD_max_ values independently predicted acute PVR in the segments other than the SAEs and SAEs (odds ratios 0.90 and 1.39 respectively). Acute PVR was not significantly associated with late atrial fibrillation recurrence.

**Conclusions:**

Avoiding PVR remains a challenge in HP‐PVI cases, but it might be resolved by setting the optimal target impedance drop and lesion distance values.

## Graphical abstract

In high‐power pulmonary vein isolation, impedance drop has a superiority to predict in‐procedure gap as compared with ablation index.

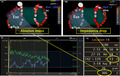

## 1 INTRODUCTION

Pulmonary vein isolation (PVI) is effective in the treatment of atrial fibrillation (AF). High‐power short‐duration (HPSD) PVI, a recently developed therapeutic option, is associated with a low radiofrequency (RF) energy requirement and short procedure and RF durations. Several studies have demonstrated acceptable acute and late outcomes in association with HPSD‐PVI.^1^, ^2^, ^3^ Unipolar signal modification (USM), a sign of transmural lesion creation by RF application, as previously described,^4^ has recently been used as a guide for HPSD‐PVI with excellent outcomes.^2^, ^5^ Despite this accumulated evidence, acute pulmonary vein reconnection (PVR) persists in 10%‐13% of patients,^1^, ^2^, ^3^ resulting in longer procedure times and larger RF energy delivery. Even when re‐ablation is performed for PVI, patients with acute PVR remain more vulnerable to late recurrence.^6^, ^7^ Accordingly, we sought to evaluate the characteristics of acute PVR following HP‐PVI under USM guidance and assess the predictors of acute PVR as surrogate markers of durability in patients with paroxysmal atrial fibrillation.

## 2 METHODS

### 2.1 Study patients

This single‐center retrospective observational study comprised 64 consecutive patients with paroxysmal AF who had undergone RF catheter ablation (RFA) using the HP‐PVI strategy from October 2018 to June 2019. Of these patients, two who were treated with the dragging technique were excluded. Three‐dimensional cardiac computed tomography (3‐D CT) with or without a contrast agent and transthoracic echocardiography were performed within a month before the procedure. Transesophageal echocardiography was performed for patients with a CHADS_2_ score ≥2 to rule out the presence of thrombi in the left atrium (LA). The use of all antiarrhythmic drugs was stopped for at least five half‐lives before the procedure. The study was approved by the institutional review board of Tokyo Women's Medical University and performed according to the institutional guidelines and in accordance with Declaration of Helsinki. All patients provided written informed consent.

### 2.2 Catheter ablation protocol

Details of the catheter ablation protocol employed have been previously published.^8^ Briefly, all patients underwent PVI and superior vena cava isolation and were deeply sedated with a 10‐minute continuous administration of dexmedetomidine (6 µg/kg/h), followed by a continuous infusion (0.3‐0.7 µg/kg/h). Four well‐trained operators performed these procedures using geometric information, as obtained from a reconstructed 3‐D CT imaging system (CARTO 3; Biosense Webster, Inc). To monitor the esophageal temperature, a multielectrode esophageal temperature‐monitoring probe was employed (Esophaster; Japan Lifeline).

**FIGURE 1:**
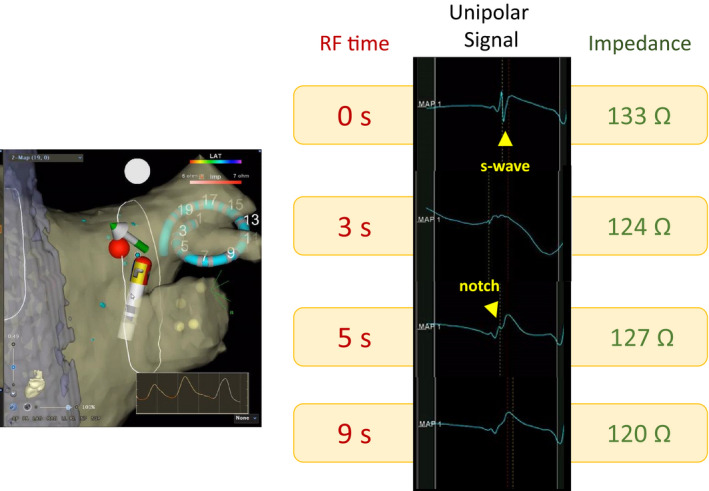
Unipolar signal modification and impedance changes. Example of a change in the unipolar electrogram result with dynamic changes in the generator impedance value. The S‐wave of unipolar signal disappears 3 s after radiofrequency catheter ablation (RFA), according to significant impedance decreases, and an R‐pattern is achieved 5 s after RFA. Following this, the impedance value gradually decreases from 133 Ω and finally reaches 120 Ω, while a notch in the positive unipolar electrogram completely disappears. RF, radiofrequency

Details of the HP‐PVI technique have been published previously.^5^ PVI was started from the right side. The disappearance of negative deflection in the unipolar electrogram recorded at the distal tip of the ablation catheter (USM; Figure 1) was adopted as an indicator of sufficient transmural necrosis.^4^, ^9^ During RFA, the CF was limited to 5‐20 g (target 10 g) for 3‐5 seconds after the USM for segments other than those adjacent to the esophagus. In case with CF >15 g or perpendicular to the atrial wall, RF was continued for only 3 seconds. RF was strictly limited to <5 seconds and CF was <10 g at the segments adjacent to the esophagus (SAEs). The target inter‐lesion distance was <5 mm. When the change of unipolar signal was unclear, we alternatively observed bipolar R‐wave decline as a surrogate indicator of lesion creation. Intensive induction of atrial overdrive pacing with isoproterenol infusion and confirmation of the absence of dormant conduction with adenosine triphosphate (ATP) infusion was attempted. This confirmation procedure was performed for at least 20 minutes after the isolation of the ipsilateral pulmonary vein (PV) pair.

### 2.3 Regional assessment of acute PVR

Acute PVR was defined as the presence of spontaneous or isoproterenol‐induced PVR or adenosine triphosphate (ATP)‐induced dormant conduction (DC) in the same session. In principle, we conventionally identified gaps (identifying the earliest electrical activation site in the antrum during sinus rhythm or pacing from the coronary sinus) and manually tagged the acute PVR sites where re‐isolation or a sequence change was observed; then, they were counted retrospectively. Late PVR was defined as PVR observed in the repeated sessions. Each LA antrum was divided into six regions, including the supero‐/infero‐anterior, supero‐/infero‐posterior, roof, and bottom regions, yielding 12 segments. In the carina, we categorized the PVR sites into four (antero‐superior, antero‐inferior, postero‐superior, and postero‐inferior) and assigned to the aforementioned 12 segments. Relevant parameters were analyzed, and the minimum values were identified for each segment as in a previous study,^10^ including the minimum contact force (CF_min_), minimum force‐time integral (FTI_min_), minimum ablation index (AI_min_), mean ablation index (AI_mean_), and minimum impedance drop (Imp‐min). The maximum inter‐lesion distance (ILD_max_) was also evaluated. Changes in the 3‐D mapping‐related indices were visualized using an on‐site monitor. Reductions in the degree of total impedance, measured every 100 ms, were monitored on a graph viewer and later exported for processing, considering the fluctuations related to the respiratory cycle. The settings of the automated ablation tag marking (VisiTag; Biosense Webster, Inc) were as previously described^5^; however, VisiTag was not utilized as a guide for the RF delivery but as one of the anatomical pieces of information on the potential site of a residual LA‐PV electrical connection after circumferential RFA around the ipsilateral PVs.

### 2.4 Follow‐up after the procedure

All patients were followed up in the outpatient clinic at 1, 3, 6, 9, and 12 months after the procedure and every 6 months thereafter. Atrial tachyarrhythmia (ATA) recurrence was evaluated according to the patients' symptoms and 24‐hour ambulatory monitoring (3, 6, 9, and 12 months after the ablation and every 6 months, thereafter). Patients with palpitations were encouraged to use a portable electrocardiographic monitoring device (HCG‐801R; Omron). Recurrence was defined as the presence of recurrent symptoms and/or detection of ATAs using the aforementioned modalities or data provided by the cardiac implantable electrical devices (ATAs lasting >30 seconds) after a 2‐month blanking period, without the use of any anti‐arrhythmic drugs.

### 2.5 Statistical analysis

Continuous variables are expressed as the mean ± standard deviation or median with interquartile range. Student's *t*‐test was used for the comparison of the characteristics of the segments with acute PVR and those without it, and the chi‐square test was used to evaluate statistical differences in the categorical variables. Regional (in the 12‐segment model) differences in the 3‐D mapping‐related indices were analyzed by analysis of variance (ANOVA). Receiver operating characteristic (ROC) curve analysis was utilized for the identification of the area under the curve (AUC) and prediction of acute PVR development for the various indices and for the determination of the cutoff value, with a specificity of 90%. Logistic regression analysis, for the evaluation of the predictors of acute PVR in the univariate and multivariate models for all segments, and region‐specific analysis were performed. Of the various 3‐D mapping‐related indices, CF_min_, FTI_min_, and AI_min_ shared the component of CF; therefore, CF_min_ and FTI_min_ were excluded from the multivariate analysis, to avoid any multicollinearity among the independent variables. A log‐rank test was performed for the Kaplan–Meier curve analysis in the assessment of the cumulative rate of ATA‐free survival of the participants. The tests were considered statistically significant at *P* < .05. All statistical analyses were performed with JMP^®^ 13 (SAS Institute Inc).

## 3 RESULTS

### 3.1 Background characteristics and clinical outcomes

Table 1 presents the 62 patients' baseline and procedural characteristics. Structural heart disease (SHD) was observed in 11 (15%) patients, and the median AF history duration was 8 months. The average procedure duration (from catheter insertion to removal) was 125 ± 46 minutes, whereas the RF duration and energy for PVI were 10 ± 3 minutes and 28 ± 8 kJ respectively. Acute PVR was observed in 44 (70%) patients; these were eliminated in the same session. All patients had undergone superior vena cava isolation after PVI. Complications occurred in only one patient; an acute right phrenic nerve injury was identified following the completion of the right‐side PVI before superior vena cava isolation. This patient showed partial recovery at the 6‐month follow‐up.

**TABLE 1:** Patients' baseline characteristics (n = 62)

Mean age (years) ± SD	62 ± 12
Male gender	47 (76)
Structural heart disease	11 (18)
HCM	4 (4)
DCM	4 (4)
ASD	2 (2)
VHD	1 (1)
Echocardiographic parameter	
LAVI (mL/m^2^)	38 ± 12
LVEF (%)	56 ± 7
History of AF [months]	8 [4‐35]
Location of esophagus	
Left	60 (96.8)
Right	1 (1.6)
Middle	1 (1.6)
Left common pulmonary vein	4 (6)
Right middle pulmonary vein	1 (2)
RF time for PVI (min) ± (SD)	10 ± 3
RF energy for PVI (kJ)	28 ± 8
Time for bilateral PVI (min)	27 ± 11
Time for the left PVI (min)	15 ± 7
Time for the right PVI (min)	12 ± 6
Total procedure time (min)	125 ± 46
Bilateral isolation length (mm)	238 ± 31
Radiation exposure (min)	10 ± 8
Additional ablation	
SVC isolation	62 (100)
CTI linear ablation	18 (29)
AT ablation	7 (11)
Non‐PV foci ablation	2 (3)

Note

Values are expressed as mean ± standard deviation, n (%), or median [interquartile range].

Abbreviations: AF, atrial fibrillation; ASD, atrial septal defect; AT, atrial tachycardia; CTI, cavo‐tricuspid isthmus; DCM, dilated cardiomyopathy; HCM, hypertrophic cardiomyopathy; LAVI, left atrial volume index; LVEF, left ventricular ejection fraction; PV, pulmonary vein; PVI, pulmonary vein isolation; RF, radiofrequency; SD, standard deviation; SVC, superior vena cava; VHD, valvular heart disease.

### 3.2 Region‐specific analysis of acute PVR

**FIGURE 2:**
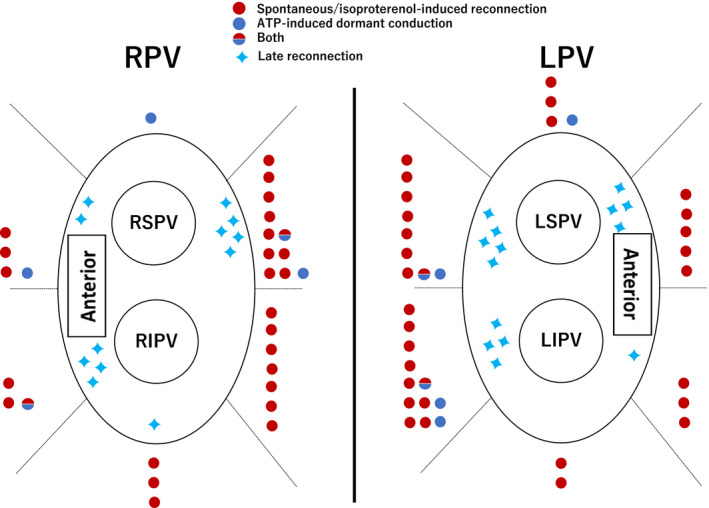
Distribution of pulmonary vein reconnection sites. The sites of acute pulmonary vein reconnection (PVR), including time‐dependent PVR and adenosine‐induced PVR, are indicated by colored circles and those of late PVR by blue four‐point stars. ATP, adenosine triphosphate; LIPV, left inferior pulmonary vein; LPV, left portal vein; LSPV, left superior pulmonary vein; RIPV, right inferior pulmonary vein; RPV, right portal vein; RSPV, right superior pulmonary vein

**FIGURE 3:**
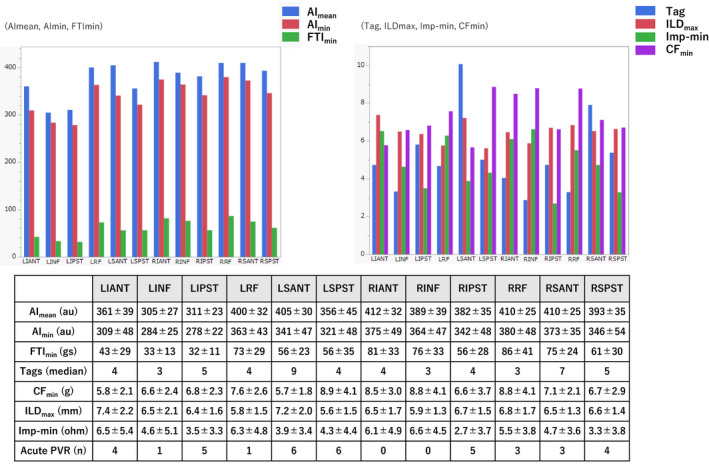
Variability in the 3‐D mapping‐related indices among each segment. The mean ablation index, minimum ablation index, minimum force‐time integral, number of tags, maximum inter‐lesion distance, minimum impedance drop, and minimum contact force values are indicated by color bars. Further details on these indices are presented in the table below. AI_mean,_ mean ablation index; AI_min_, minimum ablation index; CF_min_, minimum contact force; FTI_min_, minimum force‐time integral; ILD_max_, maximum inter‐lesion distance; Imp‐min, minimum impedance drop; LIANT, left infero‐anterior segment; LINF, left inferior segment; LIPST, left infero‐posterior segment; LRF, left roof segment; LSANT, left supero‐anterior segment; LSPST, left supero‐posterior segment; PVR, pulmonary vein reconnection; RIANT, right infero‐anterior segment; RINF, right inferior segment; RIPST, right infero‐posterior segment; RRF, right roof segment; RSANT, right supero‐anterior segment; RSPST, right supero‐posterior segment; Tag, median number of tags

Of the 1162 ablations observed in 620 segments other than the 124 SAEs, acute PVR was observed in 43 (7%) and 21 (17%) segments (*P* = .001) respectively. Acute PVR included 18 spontaneous/isoproterenol‐induced PVRs and 5 ATP‐induced DCs in the SAEs and 39 spontaneous/isoproterenol‐induced PVRs and 6 ATP‐induced DCs in segments other than SAEs. Figure 2 shows the distribution of the acute PVR sites, and Figure 3 shows the relevant indices in each segment. The degree of regional variance in these indices was significant (*P* < .0001). Spontaneous/isoproterenol‐induced PVR was more prevalent in the SAEs than in the segments other than the SAEs (15% vs 6%, *P* = .005); and for the ATP‐induced DCs, similar findings were also observed (4% vs 1%, *P* = .02). In the SAEs, significant differences were observed only in the ILD_max_ between the segments with and without acute PVR; in the segments other than the SAEs, significant differences were noted in the CF_min_, FTI_min_, AI_min_, and Imp‐min (Table 2). This difference in these indices based on the detailed classification (spontaneous/isoproterenol‐induced PVR or ATP‐induced DC) is described in Tables S1 and S2. In the ROC curve analysis, AUCs for the prediction of acute PVR absence in the SAEs and segments other than the SAEs are indicated in Figure S1. The relatively low sensitivity of each variable was evident in the discrimination of durable lesions at each known cut‐off value,^11^, ^12^, ^13^ both in the SAEs and segments other than the SAEs (Table 3). Meanwhile, durable segments were characterized by an Imp‐min of 6.5 Ω in the segments other than the SAEs, ILD_max_ of 4.8 mm in the SAEs, and AI_min_ of 405 au in the segments other than the SAEs, with a specificity of 90% (Table S3).

**TABLE 2:** Differences in the three‐dimensional mapping‐related indices in the segments with and without acute PVR

Variable	Other than the SAE			SAE		
	PVR (+) N = 43	PVR (−) N = 577	*P*‐value	PVR (+) N = 21	PVR (−) N = 103	*P*‐value
CF_min_ (g)	6.4 ± 2.7	7.4 ± 3.0	.01	6.7 ± 2.4	7.8 ± 3.5	.82
FTI_min_ (gs)	56 ± 33	67 ± 35	.02	37 ± 18	43 ± 27	.17
AI_min_ (au)	334 ± 57	350 ± 54	.02	287 ± 35	298 ± 48	.20
ILD_max_ (mm)	6.7 ± 1.8	6.6 ± 1.7	.69	6.9 ± 1.9	5.9 ± 1.4	.001
Imp‐min (Ω)	3.2 ± 4.1	5.4 ± 4.5	<.0001	3.4 ± 3.3	4.1 ± 3.8	.34

Note

Values are expressed as mean ± standard deviation.

Abbreviations: AI_min_, minimum ablation index; CF_min_, minimum contact force; FTI_min_, minimum force–time integral; ILD_max_, maximum inter‐lesion distance; Imp‐min, minimum impedance drop; PVR, pulmonary vein reconnection; SAE, segment adjacent to the esophagus.

**TABLE 3:** Predictive value of three‐dimensional mapping‐related indices at known cut‐off values

Variables	Cut‐off	Specificity	Sensitivity	PPV	NPV
Other than the SAE (n = 620)					
AI_min_	>550 au	NA	NA	NA	NA
ILD_max_	<5 mm	86	14	7	93
Imp‐min	>10 Ω	96	13	97	8
CF_min_	>10 g	96	15	98	8
FTI_min_	>400 gs	NA	NA	NA	NA
SAE (n = 124)					
AI_min_	>300 au	71	59	88	20
ILD_max_	<5 mm	86	31	91	19
Imp‐min	>10 Ω	95	6	86	17
CF_min_	>10 g	95	22	96	20
FTI_min_	>400 gs	NA	NA	NA	NA

Abbreviations: AI_min_, minimum ablation index; CF_min_, minimum contact force; FTI_min_, minimum force‐time integral; ILD_max_, maximum inter‐lesion distance; Imp‐min, minimum impedance drop; NA, not applicable; NPV, negative predictive value; PPV, positive predictive value; SAE, segment adjacent to the esophagus.

### 3.3 Predictors of acute PVR

Table S4 shows the odds ratio of acute PVR in association with the various 3‐D mapping‐related indices. In the multivariate analysis, ILD_max_ and Imp‐min were found to be the sole independent predictors of acute PVR in the SAEs and segments other than the SAEs, respectively, after adjustment for the confounders of AI_min_, ILD_max_, and Imp‐min.

### 3.4 Follow‐up and late outcomes

Over the median follow‐up duration of 12.8 months, 15 (24%) patients experienced ATA recurrence, including paroxysmal AF/atrial tachycardia (AT) in 8 (53%), persistent AF/AT in 4 (27%), and antiarrhythmic drug use without any arrhythmia occurrence in 3 (20%) (treated as a recurrence) patients. The ATA‐free rates were 81% and 74% at 6 months and 1 year after the procedure, respectively; the exclusion of patients with SHD increased these values to 90% and 86% at 6 months and 1 year after the procedure respectively. There was no significant difference in the occurrence of late outcomes between the patients with and without acute PVR (*P* = .64; Figure S2). Of patients with late recurrence, 12 underwent a redo procedure a median of 203 days after the first session. Ten patients had segments with late PVR (Figure 2) (nine [38%] SAEs vs 17 [14%] segments other than the SAEs, *P* = .01). Only three segments with late PVR matched those with acute PVR. There was no significant difference in the 3‐D mapping‐related indices between the segments with late PVR and those without it. Only the Imp‐min tended to be higher in the segments other than the SAEs without late PVRs than in those with them (4.7 ± 4.3 vs 2.7 ± 3.3, *P* = .09); this was not observed in the other indices.

## 4 DISCUSSION

In the present study, HP‐PVI was achieved under USM guidance with relatively short procedure and isolation durations, and acceptable late outcomes, among the patients with paroxysmal AF. The AI_min_ value required for acute PVR avoidance was remarkably lower than that used in clinical practice^1^; in addition, higher ILD_max_ and lower Imp‐min values were shown to be independently predictive of acute PVR during the procedure in the SAEs and segments other than the SAEs, respectively, after adjusting for AI_min_. Acute PVR was not significantly associated with late AF recurrence.

Recent studies have reported the superior efficacy and safety of HPSD‐PVI compared to conventional PVI,^14^, ^15^ showing that the RF time and energy can be reduced with AF‐free survival maintenance.^2^, ^3^ Theoretically, HPSD‐PVI can reduce the degree of collateral damage with shallow and wide lesion creation through resistive heating.^16^, ^17^ Additionally, several studies have demonstrated that the results of HPSD‐PVI are noninferior to those observed following conventional PVI, in terms of clinical safety.^18^, ^19^ In the present study, only one patient with an enlarged LA experienced acute right phrenic nerve injury after right‐sided PVI and partially recovered within half a year. Subsequently, a 10‐mA stimulation test was routinely performed at the right superior PV antrum for the avoidance of phrenic nerve injury. Esophageal injury after RF application is one of the major complications in PVI; therefore, RF application should be gently and carefully performed in SAEs. The present study demonstrated ILD_max_ was a significant predictor of PVR in the SAEs among the various indices, which was partially in line with the findings of a previous report^20^ showing the optimal indicator of ILD_max_ for the prediction of PVR in the left posterior wall even if AI value was sufficiently obtained (not high‐power strategy). Recent literature revealed that a strong contact and high AI value were associated with the severity of esophageal injury in the HP‐PVI.^21^ Based on these results, SAEs clearly should be treated, considering the tightened ILD with a modest contact force.

Several attempts have been made to increase patients' ATA‐free survival rates, including the use of various guidance methods that are performed during PVI (CF, FTI, AI, or impedance drop), or the confirmation of acute PVR using isoproterenol infusion or ATP after PVI, which is considered responsible for durable lesion creation. Most importantly, all the acute PVRs were eliminated by a touch‐up procedure in the same session, and the waiting time (20 minutes) after the final ipsilateral PVI was sufficient, rendering the lesion creation robust. Additionally, the acute and late reconnection sites were not equivalent.^22^ Nevertheless, converse advocation has been noted in the past. Efremidis et al demonstrated the presence of superior late outcomes in patients without acute PVRs, as confirmed 30 minutes after the final application.^6^ Anter et al concluded that acute PVR is associated with six times the risk of late AF recurrence even when appropriately eliminated.^7^ This can be attributed to the existence of multiple vulnerable sites. Although this theme needs further investigation, acute PVR may be substantially associated with longer RF durations and larger rates of RF energy delivery, leading to tissue overheating.

**FIGURE 4:**
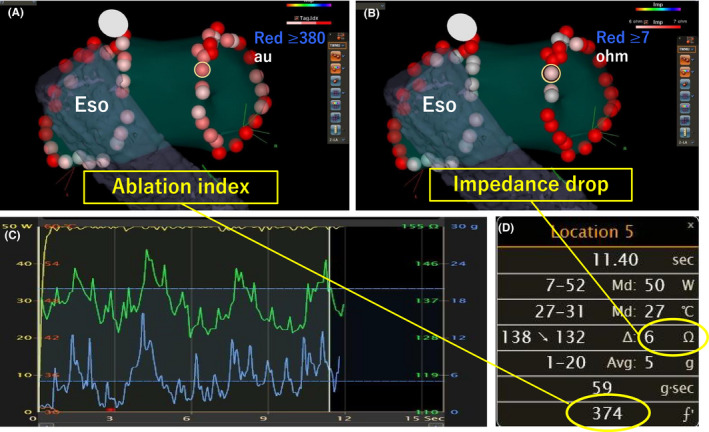
Representative figure for relevant parameters in an acute PVR site. On posterior‐anterior projection, VisiTags indicate the ablation index (A) and impedance drop (B) of each lesion. The acute pulmonary vein reconnection (PVR) site is indicated by a yellow circle at the right supero‐posterior segment. Dynamic changes in the impedance and contact force values, as observed in Graph viewer, are shown in green and blue respectively. Fluctuation in the impedance values is observed (C). Parameters corresponding to the acute PVR site (D). Eso, esophagus

**FIGURE 5:**
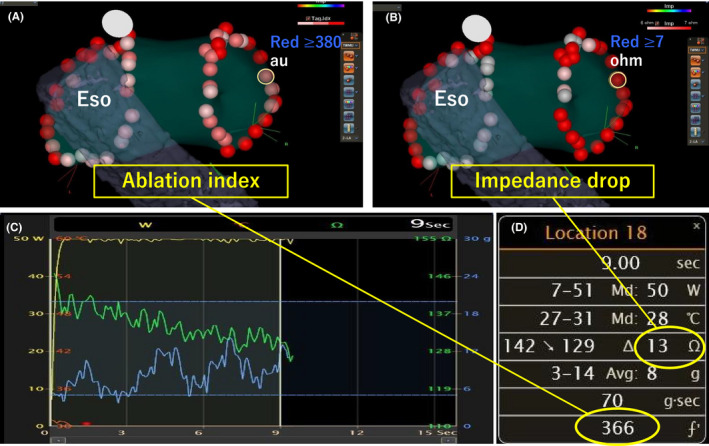
Representative figure for relevant parameters in a site without acute PVR. On posterior‐anterior projection, VisiTags indicate the ablation index (A) and impedance drop (B) values of each lesion. The site without acute pulmonary vein reconnection (PVR) is indicated by a yellow circle at the supero‐anterior segment. Dynamic changes in the impedance and contact force values, as observed in Graph viewer, are shown in green and blue respectively. Gradual and stable decreases in the impedance values are observed (C). Parameters corresponding to these sites (D). Eso, esophagus

Although the AI is a reliable indicator of lesion creation in PVI at a conventional power and duration, it is unclear whether it is also suitable in HPSD‐PVI. Previous studies have reported the efficacy of HPSD‐PVI guided by the AI and lesion size index (LSI).^1^, ^23^, ^24^ However, neither the AI nor LSI could directly exacerbate tissue damage. Furthermore, since CF is dependent on the contact sensor at the distal tip of the ablation catheter, sensor errors occur often. Indeed, the AI_min_ required for the prevention of acute PVR was remarkably low in the present study compared to that applied previously.^1^, ^23^ In contrast, a high Imp‐min value was independently predictive of acute PVR absence; furthermore, relatively low values (6.5 Ω) were needed for durability. Figures 4 and 5 present a comparison of the VisiTags of the AI and impedance drop values in segments with and without acute PVR respectively. The AI_min_ value was almost the same, regardless of the presence of an acute PVR site; meanwhile, impedance drop clearly enhanced that with an insufficient decrease and non‐negligible respiratory fluctuations. These findings imply that the AI has lower reliability than impedance drop in HP‐PVI.

Several studies have highlighted the significance of impedance drop in durable lesion creation.^11^, ^25^ USM may directly enhance the rate of cellular necrosis without any collateral damages; additionally, impedance drop has shown similar characteristics in both experimental and clinical settings.^9^, ^11^, ^26^ Figure 1 presents an example of a durable site in the procedure, demonstrating that the R‐wave pattern in the unipolar signal was immediately (5 seconds) achieved after RFA and reached a plateau; however, the generator impedance value gradually decreased, finally reaching a 13‐Ω decrease 9 seconds after RFA. This continuous decline in the generator impedance value after USM had a substantial impact on durable lesion creation.^9^ Occasionally, unipolar signals may not be visible because of electrical artifacts; this weakness may be overcome with the use of impedance drop guidance. However, impedance change itself is strongly affected by catheter contact^27^ and contact angle^28^; the VisiTag of impedance drop is reflected a few seconds after the absolute impedance decrease because of its relatively long calculation time. These points are major obstacles in using this PVI indicator onsite. Nevertheless, re‐ablating any sites where there is an impedance drop <6.5 ohms after the first encircling have a potential to reduce PVRs although this is not an onsite strategy. This may be helpful for not only identifying the gap site but also avoiding insufficient lesion creation. Recently, real‐time local impedance decrease was used as a target of durable lesion creation using a dedicated catheter,^29^, ^30^ which may facilitate the achievement of impedance‐guided HP‐PVI with less PVRs.

There are certain limitations to this study. First, our study had a relatively small sample size. Although this may have led to the underpowering of the statistical analysis, in terms of per‐segments analysis, 744 regions were considered, which, in turn, could improve the level of statistical confidence. Second, Imp‐min and late recurrence had an associative tendency, but this association was not significant in the segments other than the SAEs, which may be derived from a small number of participants for statistics. Lastly, the acute PVRs were distributed heterogeneously. Future studies with larger sample sizes should assess each segment for the clarification of regional differences in the characteristics of acute PVR.

## 5 CONCLUSIONS

Our findings primarily suggest that in association with USM‐guided HP‐PVI, lesion distance and impedance drop are better predictors of acute PVR in the SAEs and segments other than the SAEs, respectively, as compared with other 3‐D mapping‐related indices.

## CONFLICT OF INTEREST

Authors declare no conflict of interests for this article.

## Supporting information

Supplementary Material
