# Supplementary material for: Regional differences in the predictors of acute electrical reconnection following high‐power pulmonary vein isolation for paroxysmal atrial fibrillation
Source: J Arrhythm. 2021 Jul 23;37(5):1260–9. doi: 10.1002/joa3.12597 (PMC8485794; doi:10.1002/joa3.12597)
Supplement: Supplementary file 1 — Supplementary Material [file JOA3-37-1260-s001.docx]

**Online Figure 1. Receiver operating characteristic curve analysis for durable lesion prediction**

Receiver operating characteristic curve analysis revealed the AUC for the prediction of durable sites in the SAE (A) and the segments other than the SAE (B). AUC values are delineated on the right side among the various 3-D mapping-related indices. AI_min_, minimum ablation index; AUC, area under the curve; CF_min_, minimum contact force; FTI_min_, minimum force–time integral; ILD_max_, maximum inter-lesion distance; Imp-min, minimum impedance drop; SAE, segment adjacent to the esophagus; 3-D, three-dimensional
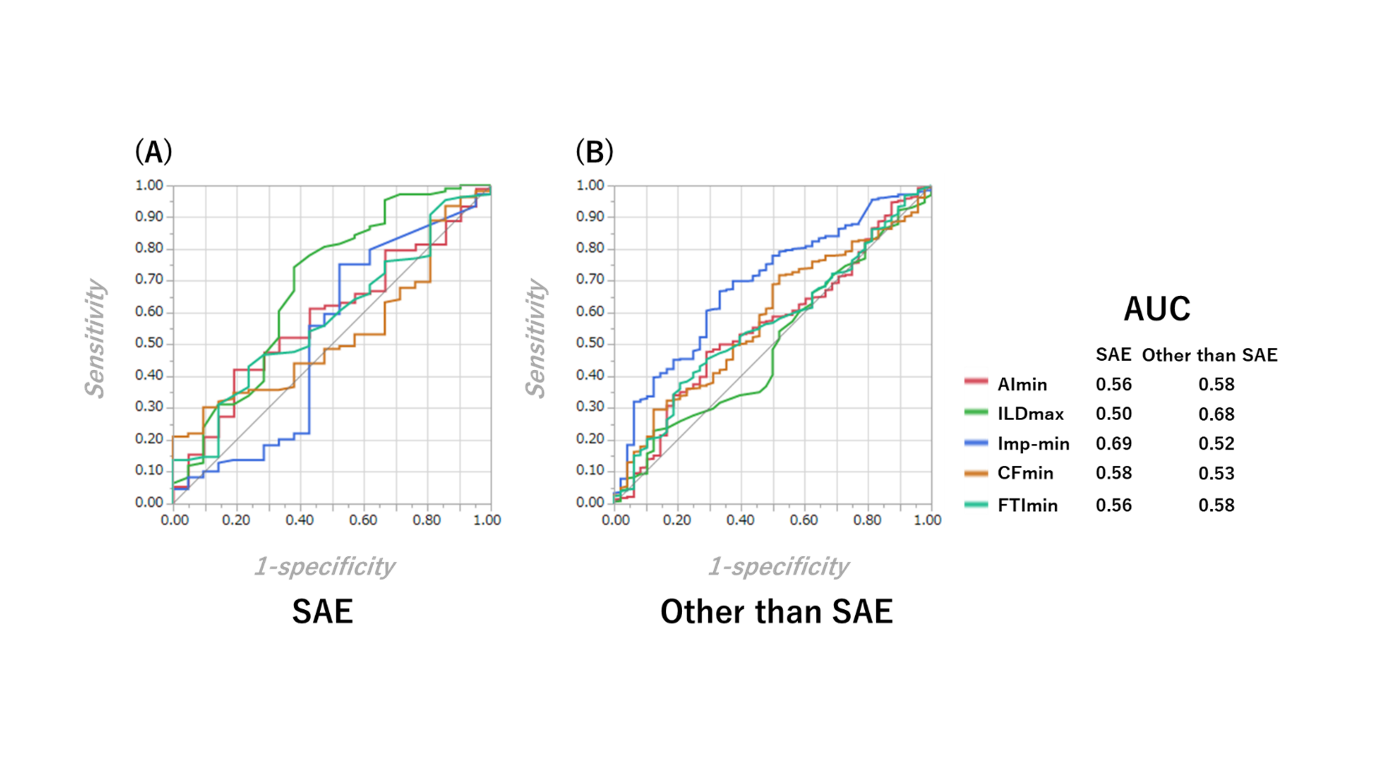


**Online Figure 2. The difference in late recurrence according to acute pulmonary vein isolation**

Kaplan-Meier curve analysis revealed no significant difference in late recurrence rate between patients with and without acute pulmonary vein reconnection (PVR).


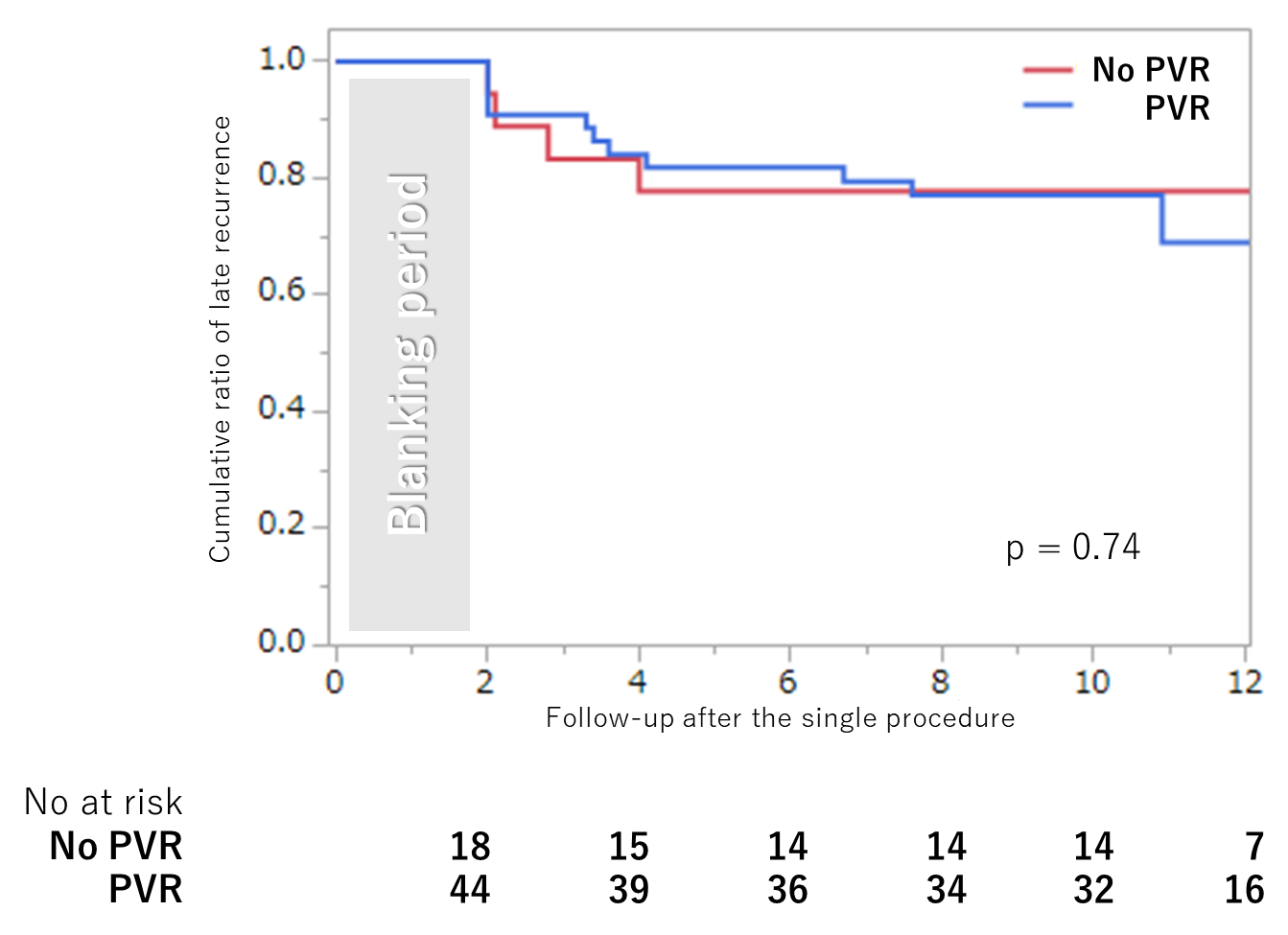


**Online Table 1. Differences in the three-dimensional mapping-related indices in the segments with and without spontaneous/isoproterenol-induced PVR**

| Variable | Other than the SAE | | | SAE | | |
| --- | --- | --- | --- | --- | --- | --- |
|  | PVR (+) | PVR (−) | p-value | PVR (+) | PVR (−) | p-value |
|  | N = 39 | N = 581 |  | N = 18 | N = 106 |  |
| CF_min_ (g) | 6.4 ± 2.4 | 7.3 ± 3.2 | 0.09 | 7.0 ± 1.9 | 8.0 ± 3.7 | 0.30 |
| FTI_min_ (gs) | 55 ± 29 | 65 ± 34 | 0.09 | 39 ± 16 | 45 ± 30 | 0.37 |
| AI_min_ (au) | 336 ± 55 | 348 ± 53 | 0.17 | 293 ± 32 | 301 ± 45 | 0.48 |
| ILD_max_ (mm) | 6.4 ± 1.3 | 6.6 ± 1.8 | 0.49 | 7.3 ± 2.4 | 5.8 ± 1.3 | < 0.001 |
| Imp-min (Ω) | 2.8 ± 3.7 | 5.2 ± 4.5 | 0.002 | 4.0 ± 3.8 | 3.9 ± 3.9 | 0.93 |

Values are expressed as mean ± standard deviation.

AI_min_, minimum ablation index; CF_min_, minimum contact force; FTI_min_, minimum force–time integral; ILD_max_, maximum inter-lesion distance; Imp-min, minimum impedance drop; PVR, pulmonary vein reconnection; SAE, segment adjacent to the esophagus

**Online Table 2. Differences in the three-dimensional mapping-related indices in the segments with and without ATP-induced DC**

| Variable | Other than the SAE | | | SAE | | |
| --- | --- | --- | --- | --- | --- | --- |
|  | DC (+) | DC (−) | p-value | DC (+) | DC (−) | p-value |
|  | N = 6 | N = 614 |  | N = 5 | N = 119 |  |
| CF_min_ (g) | 6.6 ± 1.3 | 7.2 ± 3.2 | 0.64 | 6.6 ± 1.7 | 7.9 ± 3.5 | 0.43 |
| FTI_min_ (gs) | 64 ± 15 | 64 ± 34 | 0.99 | 35 ± 21 | 45 ± 29 | 0.46 |
| AI_min_ (au) | 361 ± 27 | 347 ± 54 | 0.53 | 280 ± 44 | 301 ± 43 | 0.29 |
| ILD_max_ (mm) | 7.5 ± 0.7 | 6.6 ± 1.7 | 0.21 | 5.7 ± 0.6 | 6.0 ± 1.6 | 0.66 |
| Imp-min (Ω) | 1.0 ± 3.6 | 5.1 ± 4.5 | 0.03 | 1.4 ± 1.9 | 4.0 ± 3.9 | 0.15 |

Values are expressed as mean±standard deviation.

AI_min_, minimum ablation index; ATP, adenosine triphosphate, CF_min_, minimum contact force; FTI_min_, minimum force–time integral; ILD_max_, maximum inter-lesion distance; Imp-min, minimum impedance drop; DC, dormant conduction; SAE, segment adjacent to the esophagus

**Online Table 3. Three-dimensional mapping-related indices indicative of the absence of acute PVR with a specificity of 90%**

| Variable | SAE | Other than the SAE | Entire |
| --- | --- | --- | --- |
| AI_min_ (au) | 334 | 405 | 383 |
| ILD_max_ (mm) | 4.8 | 8.1 | 4.8 |
| Imp-min (Ω) | 8.5 | 6.5 | 7.3 |

AI_min_, minimum ablation index; ILD_max_, maximum inter-lesion distance; Imp-min, minimum impedance drop; PVR, pulmonary vein reconnection; SAE, segment adjacent to the esophagus.

**Online Table 4. Predictors of acute PVR**

| Variable | Other than the SAE | | | SAE | | | |  |
| --- | --- | --- | --- | --- | --- | --- | --- | --- |
|  | OR | 95% CI | p-value | | OR | 95% CI | p-value | |
| Univariate analysis | | | | | | | |  |
| AI_min_ (au) | 0.99 | 0.99–1.00 | 0.02 | | 0.99 | 0.99–1.00 | 0.21 | |
| ILD_max_ (mm) | 1.02 | 0.89–1.18 | 0.69 | | 1.42 | 1.13–1.78 | 0.002 | |
| Imp-min (Ω) | 0.89 | 0.83–0.94 | < 0.0001 | | 0.95 | 0.85–1.06 | 0.34 | |
| CF_min_ (g) | 0.88 | 0.79–0.97 | 0.01 | | 0.89 | 0.78–1.02 | 0.08 | |
| FTI_min_ (gs) | 0.99 | 0.98–0.99 | 0.01 | | 0.99 | 0.97–1.01 | 0.17 | |
| Multivariate analysis | | | | | | | |  |
| AI_min_ (au) | 0.99 | 0.99–1.00 | 0.19 | | 0.99 | 0.98–1.01 | 0.21 | |
| ILD_max_ (mm) | 1.01 | 0.88–1.17 | 0.87 | | 1.39 | 1.11–1.74 | 0.005 | |
| Imp-min (Ω) | 0.90 | 0.85–0.95 | 0.0003 | | 0.97 | 0.87–1.08 | 0.53 | |

AI_min_, minimum ablation index; CF_min_, minimum contact force; CI, confidence interval; FTI_min_, minimum force–time integral; ILD_max_, maximum inter-lesion distance; Imp-min, minimum impedance drop; OR, odds ratio; PVR, pulmonary vein reconnection; SAE, segment adjacent to the esophagus
